# Silk fibroin promotes H3K9me3 expression and chromatin reorganization to regulate endothelial cell proliferation

**DOI:** 10.1063/5.0203858

**Published:** 2024-05-29

**Authors:** Kaixiang Gao, Yafan Xie, Fangning Xu, Qin Peng, Li Fu, Guixue Wang, Juhui Qiu

**Affiliations:** 1 Key Laboratory for Biorheological Science and Technology of Ministry of Education, State and Local Joint Engineering Laboratory for Vascular Implants, Bioengineering College of Chongqing University, Chongqing 400030, China; 2 Institute of Systems and Physical Biology, Shenzhen Bay Laboratory, Shenzhen 518132, China; 3 The Eighth Affiliated Hospital, Sun Yat-sen University, Shenzhen 518033, China; 4 Jinfeng Laboratory, Chongqing 401329, China

## Abstract

Silk fibroin (SF), which is extensively utilized in tissue engineering and vascular grafts for enhancing vascular regeneration, has not been thoroughly investigated for its epigenetic effects on endothelial cells (EC). This study employed RNA sequencing analysis to evaluate the activation of histone modification regulatory genes in EC treated with SF. Subsequent investigations revealed elevated H3K9me3 levels in SF-treated EC, as evidenced by immunofluorescence and western blot analysis. The study utilized H2B-eGFP endothelial cells to demonstrate that SF treatment results in the accumulation of H2B-marked chromatin in the nuclear inner cavities of EC. Inhibition of H3K9me3 levels by a histone deacetylase inhibitor TSA decreased cell proliferation. Furthermore, the activation of the MAPK signaling pathway using chromium picolinate decreased the proliferative activity and H3K9me3 level in SF-treated EC. SF also appeared to enhance cell growth and proliferation by modulating the H3K9me3 level and reorganizing chromatin, particularly after oxidative stress induced by H_2_O_2_ treatment. In summary, these findings indicate that SF promotes EC proliferation by increasing the H3K9me3 level even under stress conditions.

## I. INTRODUCTION

Silk fibroin (SF) serves as a prominent scaffold matrix within the field of tissue engineering and regenerative medicine. It is distinguished by its remarkable mechanical properties, excellent compatibility with cells, customizable biodegradability, ease of fabrication, and adjustable processing parameters.^1–5^ SF can facilitate cell attachment and proliferation in various cell types, both *in vitro* and *in vivo*.^3,4,6–8^ Furthermore, SF has proven effective in therapeutic applications aimed at expediting vascular regeneration by promoting the growth of endothelial cells (EC).^1^ Nevertheless, the challenge in vascular tissue engineering lies in obtaining clinically significant quantities of EC, given their limited proliferative capacity and low isolation yields.

Chromatin, comprising histones, DNA, and nonhistone proteins, plays a pivotal role in regulating gene expression, thereby affecting all DNA-templated activities and key biological processes, including cell proliferation and reprogramming.^9^ The organization of chromatin is subject to regulation by multiple factors that influence gene expression.^10–13^ For instance, histone methylation, exemplified by trimethylated histone H3 at lysine 9 (H3K9me3) and trimethylated histone H3 at lysine 27 (H3K27me3), reduces chromatin accessibility, resulting in decompaction and transcriptionally inactive states. Notably, the well-established epigenetic marker H3K9me3 is expressed at high levels in genes related to the cell cycle during progression.^14–16^ Additionally, inhibiting H3K9me3 disrupts chromatin reorganization and transcription of cardiac genes.^10^ Functional analyses revealed the tight regulation of H3K9me3 in a cell cycle-dependent manner and its role in chromatin reorganization. However, the specific histone modifications and associated chromatin reorganization mechanisms involved in SF-induced EC proliferation and vascular regeneration have not been fully elucidated.

We hypothesized that histone modifications and chromatin reorganization constitute the principal mechanisms underlying SF-induced EC proliferation. Leveraging RNA-seq and H2B-eGFP transgenic EC, our investigation revealed that SF-treated cells exhibit an accumulation of chromatin marked by H2B in the inner cavities of the nucleus. Furthermore, we demonstrated that the suppression of H3K9me3 with trichostatin A (TSA) impedes cell proliferation. For upstream signaling, we discovered that the MAPK pathway for SF induced EC proliferation with the MAPK activator chromium picolinate. Additionally, in the presence of oxidative stress, SF enhances cell survival through chromatin reorganization and H3K9me3 modification. These findings collectively suggest that SF induces chromatin reorganization and H3K9me3 modification, suggesting novel therapeutic avenues for the treatment of vessel regeneration.

## II. RESULTS

### A. SF solution promotes endothelial cell proliferation

**FIG. 1.**
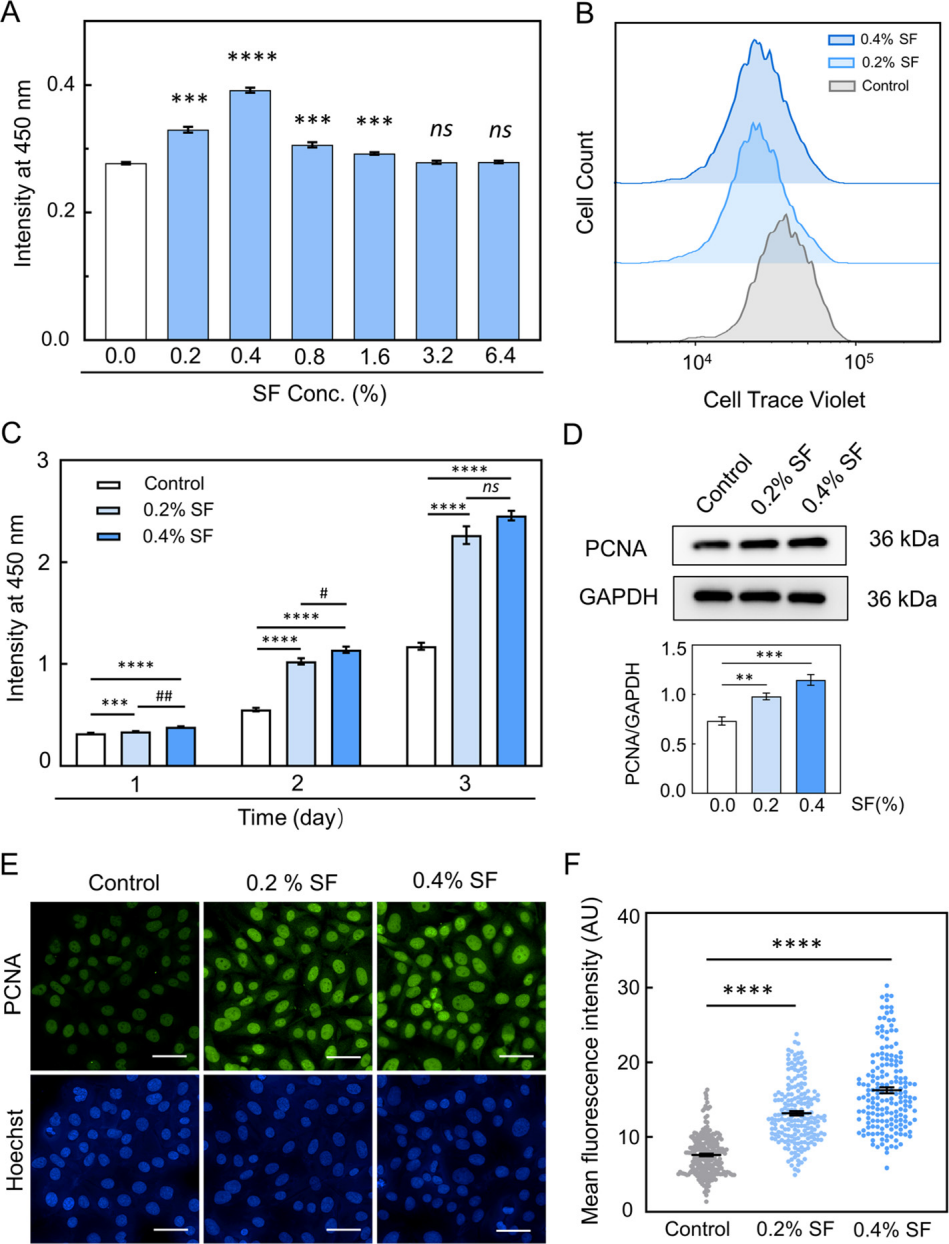
SF promotes endothelial cell proliferation. (a) HUVECs were treated with the indicated concentration of SF for 24 h, and cell proliferation was visualized via a CCK-8 assay. (b) Histogram plots of cell trace intensity gated on HUVECs that were cultured for 2 days with SF. (c) Viability of HUVECs after treatment with SF for 1, 2, or 3 days. (d) Western blot analysis was performed to detect PCNA expression in HUVECs treated with 0.2% or 0.4% SF solution for 2 days. (e) Immunofluorescence staining of PCNA in HUVECs treated with 0.2% or 0.4% SF solution for 2 days (scale bar = 50 *μ*m). Hoechst was used as a DNA counterstain. (f) The mean fluorescence intensity of PCNA was further compared (n > 150). The mean ± SEM is presented based on more than three separate experiments. Compared with control, statistical significance is indicated by ^*^p ≤ 0.05, ^**^p ≤ 0.01, ^***^p ≤ 0.001, and ^****^p ≤ 0.0001. Compared with the 0.2% SF-treated group, statistical significance is indicated by #p ≤ 0.05. The “ns” indicates no statistical significance. The statistical significance was estimated using a two-tailed t-test.

To determine the optimal concentration of SF solution for promoting proliferation, we initially conducted a CCK-8 assay. Our findings revealed that SF induced cell growth in a dose-dependent manner within the concentration range of 0.2%–1.6%, with 0.4 (wt. %/vol. %) SF exhibiting the most pronounced effect on EC proliferation [Fig. 1(a)]. Furthermore, higher concentrations (3.2% and 6.4%) of SF solution did not significantly differ from those in the control group. We also observed that SF increased mouse brain endothelial bEnd.3 cell proliferation with concentrations of 0.2% and 0.4% (Fig. S1).

Consequently, based on these results, we selected 0.2% and 0.4% SF for subsequent experiments. Additionally, we explored the time-dependent impact of SF treatment on HUVECs using concentrations of 0.2% and 0.4% over 3 days. The proliferative capacity of HUVECs treated with 0.2% and 0.4% SF increased nearly linearly over time [Fig. 1(c)]. By day 3, the proliferation induced by 0.2% and 0.4% SF became comparable, possibly due to the cells reaching complete confluence and experiencing further growth inhibition.

CellTrace Violet dye is widely utilized for assessing cell proliferation directly.^17^ To investigate the HUVECs division, we labeled the cells with CellTrace Violet dye and treated them with 0.2% and 0.4% SF for 2 days. Flow cytometry analysis revealed that SF treatment significantly enhanced HUVEC proliferation [Fig. 1(b)]. Proliferating cell nuclear antigen (PCNA) plays a pivotal role in DNA replication and cell cycle regulation.^18^ Therefore, we compared the PCNA protein level in HUVECs treated with SF for 2 days. The expression level of PCNA was markedly elevated upon SF treatment [Figs. 1(d)–1(f)]. We also examined whether SF influenced cell migration through a scratch assay using concentrations of 0.2% and 0.4%. However, no noticeable improvement in migration was observed with any SF concentration in both HUVECs and bEnd.3 cells (Figs. S2 and S3). These findings confirmed that SF promoted endothelial cell growth and proliferation.

### B. SF treatment increases the level of H3K9me3 in ECs

**FIG. 2.**
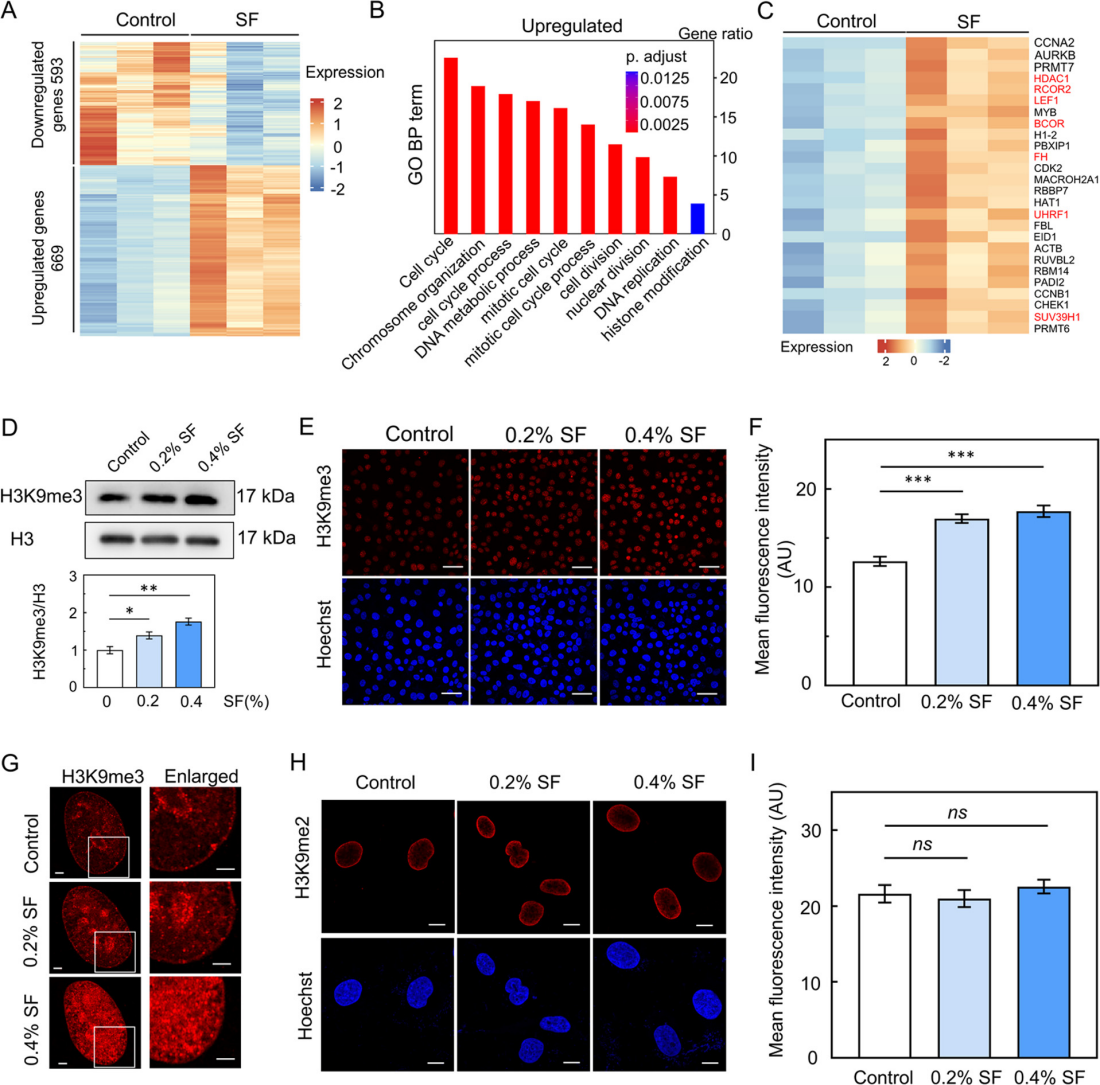
SF treatment increases the level of H3K9me3 in ECs. (a) Heatmap showing the changes in gene expression in the SF group (n = 3) compared with the control group (n = 3). The expression of significantly upregulated (n = 669, red) or downregulated (n = 593, blue) genes (adjusted P value < 0.05, FC ≥ 1.2) is indicated. The legend for the color scale is shown. (b) Bar plot of GO biological process terms for the significantly upregulated genes. (c) Heatmap showing that 26 genes related to histone modification were upregulated under SF treatment conditions. (d) Western blot analysis was performed to detect H3K9me3 levels in HUVECs treated with 0.2% or 0.4% SF solution for 2 days. (e) Immunofluorescence staining was used to detect H3K9me3 levels in control and SF-treated HUVECs after 2 days (scale bar = 50 *μ*m). Hoechst was used as a DNA counterstain. (f) The mean fluorescence intensity of H3K9me3 was compared (n > 150). (g) The observed reorganization of H3K9me3-marked chromatin in the SF groups (scale bar = 2 *μ*m). (h) HUVECs were treated with 0.2% or 0.4% SF solution for 2 days and stained for H3K9me2 (scale bar = 10 *μ*m). Hoechst was used as a DNA counterstain. (i) The mean fluorescence intensity of H3K9me2 was compared (n > 150). The mean ± SEM is presented based on more than three separate experiments. Statistical significance is indicated by ^*^p ≤ 0.05, ^**^p ≤ 0.01, and ^***^p ≤ 0.001, while '*ns*' indicates no statistical significance.

To determine the differences in gene expression between SF-treated HUVECs and controls, we conducted a comparative RNA sequencing (RNA-seq) analysis of HUVECs exposed to 0.4% SF for 2 days. Differential expression analysis revealed 593 downregulated and 669 upregulated genes (cutoff: adjusted P value < 0.05, fold change ≥ 1.2) [Fig. 2(a)]. Downregulated genes are represented in blue, while upregulated genes are represented in red. To explore the differences in gene expression between the two culture conditions, we conducted the Kyoto Encyclopedia of Genes and Genomes (KEGG) pathway enrichment analysis, which revealed a preference for the upregulation of multiple cell cycle pathways in SF-cultured cells (Fig. S4). Additionally, gene ontology (GO) term analysis demonstrated that, compared with the control treatment, SF treatment led to the upregulation of genes associated with cell cycle progression [Fig. 2(b)].

Subsequently, we leveraged our RNA-seq data to identify gene expression changes related to chromosome organization and histone modification. Notably, SF treatment primarily increased the expression of H3K9me3-related genes, including HDAC1, EGR1, UHRF1, RCOR2, and SUV39H1 [Figs. 2(c) and S5]. As a result, we hypothesized that SF treatment would elevate H3K9me3 levels and contribute to cell proliferation. To validate this hypothesis, HUVECs were treated with DPBS, 0.2% SF, or 0.4% SF for 2 days. Western blot analysis revealed that SF treatment upregulated H3K9me3 levels [Fig. 2(d)]. In parallel, HUVECs treated with SF exhibited a significantly greater H3K9me3 level [Figs. 2(e) and 2(f)]. SF solution treatment also increased the H3K9me3 level in mouse brain endothelial bEnd.3 cells (Fig. S6).

Moreover, cells treated with SF displayed an accumulation of H3K9me3-modified chromatin within the nuclear inner cavities on day 2 compared to that in the control group [Fig. 2(g)]. Furthermore, immunofluorescence staining analysis of HUVECs following 2 days of SF treatment revealed no significant difference in the level of H3K9me2 compared to those in the control groups [Figs. 2(h) and 2(1)]. In summary, these findings suggest that the upregulation of H3K9me3 in EC following SF treatment may be linked to cell proliferation.

### C. H3K9me3 is responsible for SF-induced EC proliferation

**FIG. 3.**
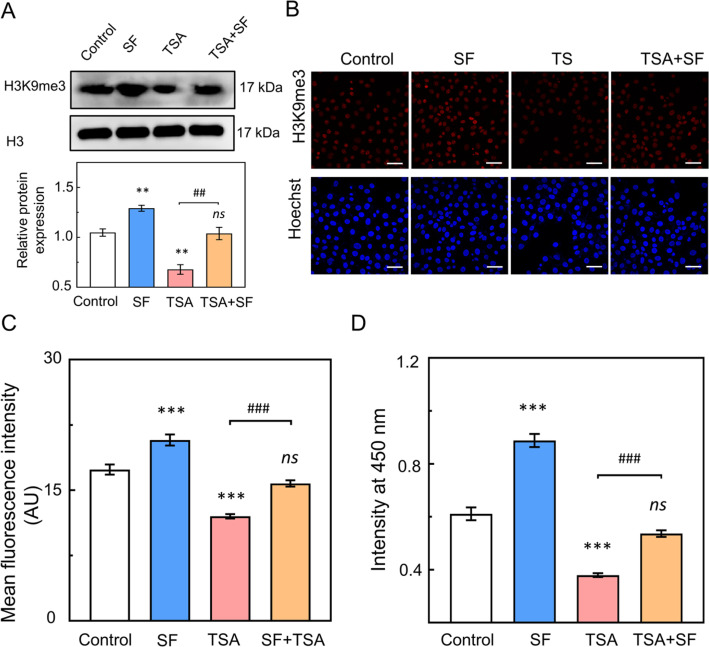
H3K9me3 is responsible for SF-induced EC proliferation. (a) Western blotting was used to detect the expression levels of H3K9me3 in the control, SF, TSA, and TSA treated with SF groups. (b) Immunofluorescence staining was used to detect the level of H3K9me3 in the control, SF, TSA, and TSA-treated SF groups (scale bar = 50 *μ*m). (c) The fluorescence intensity of H3K9me3 in each group was analyzed and compared (n ≥100). (d) Proliferation of HUVECs cultured with DMSO, SF, TSA, or TSA and treated with SF for 2 days. The means ± SEM are presented based on more than three separate experiments. Compared with control, statistical significance is indicated by ^*^p ≤ 0.05, ^**^p ≤ 0.01, and ^***^p ≤ 0.001. Compared with the TSA-treated group, statistical significance is indicated by ##p ≤ 0.01 and ###p ≤ 0.001. The “ns” indicates no statistical significance. The statistical significance was estimated using a two-tailed t-test. The statistical significance was estimated using a two-tailed t-test.

To investigate whether the increase in H3K9me3 influenced the proliferation of HUVECs in the SF-treated group, we assessed the impact of reducing H3K9me3 levels using TSA, a small molecule inhibitor of histone deacetylase (HDAC).^19^ HUVECs were exposed to 0.4% SF, 100 nM TSA, or a combination of 100 nM TSA with 0.4% SF for two days. The negative control sample was treated with DMSO. Western blot analysis was also conducted under identical conditions, revealing that TSA reduced the level of H3K9me3 in a manner consistent with the pattern observed in SF-induced H3K9me3 [Fig. 3(a)]. Subsequently, we performed immunofluorescence staining and quantified the mean fluorescence intensity of H3K9me3. The levels of H3K9me3 were greater in the TSA-treated group treated with SF than in the TSA-treated group [Figs. 3(b) and 3(c)]. Treatment with both TSA and SF resulted in increased cell proliferation compared to treatment with TSA alone [Fig. 3(d)]. These results strongly suggest that SF treatment leads to an increase in the level of H3K9me3, subsequently promoting cell proliferation.

### D. SF regulates the reorganization of nuclear chromatin

**FIG. 4.**
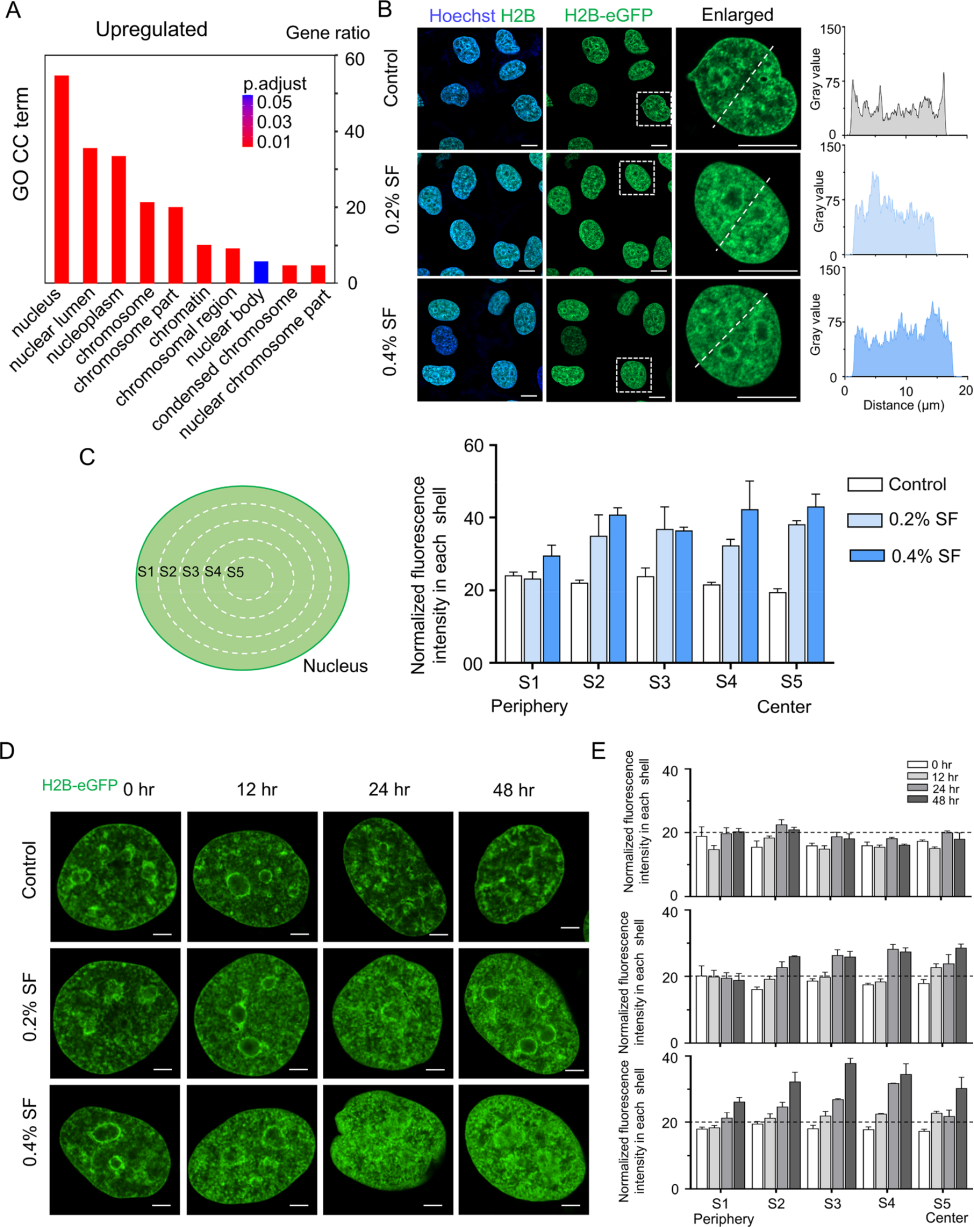
SF regulates the reorganization of nuclear chromatin. (a) GO cellular component term analysis was conducted for the upregulated genes. (b) H2B-eGFP transgenic HUVECs were treated with 0.2% or 0.4% SF solution for 48 h (scale bar = 10 *μ*m). The H2B concentration along the white line was analyzed via ImageJ software. H2B-marked overall chromatin was highly localized at the nuclear center and formed large chromatin domains in the SF groups. (c) Normalized fluorescence intensity of H2B-eGFP in each shell in control (n = 31) and SF-treated cells (n = 32). (d) SF regulates the reorganization of nuclear chromatin. H2B-eGFP transgenic HUVECs were cultured with 0.2% or 0.4% SF solution for 0, 12, 24, or 48 h (scale bar = 2 μm). (E) Normalized fluorescence intensity of H2B-eGFP in each shell in control (n = 36) and SF-treated cells (n = 34).

Notably, our analysis revealed that, according to the GO cellular component term, genes related to nuclear components were predominantly upregulated, including the nucleus, nucleoplasm, chromosome, and condensed chromosome [Fig. 4(a)]. Furthermore, the GO molecular functional term analysis demonstrated that SF treatment induced the upregulation of genes associated with nuclear transcriptional regulation, such as genes related to nucleoside-triphosphatase activity, chromatin binding, and histone binding (Fig. S7). To assess chromatin distribution, we established an H2B-eGFP HUVEC line and treated the cells with DPBS, 0.2%, or 0.4% SF for 2 days. As anticipated, cells treated with SF exhibited an accumulation of H2B-marked overall chromatin at the nuclear inner cavities on day 2, in contrast to the control group [Figs. 4(b) and 4(c)]. Additionally, compared with those of the control, the morphology of HUVECs treated with SF exhibited distinct changes, as indicated by a larger nuclear area (Fig. S8). Furthermore, compared with cells in the control group, cells treated with SF demonstrated an accumulation of H2B-marked overall chromatin within the nuclear inner cavities from 0 to 48 h [Figs. 4(d) and 4(e)]. In summary, these results illustrate that SF treatment can influence the organization of chromatin.

### E. SF promotes EC proliferation through downregulation of the MAPK signaling pathway

**FIG. 5.**
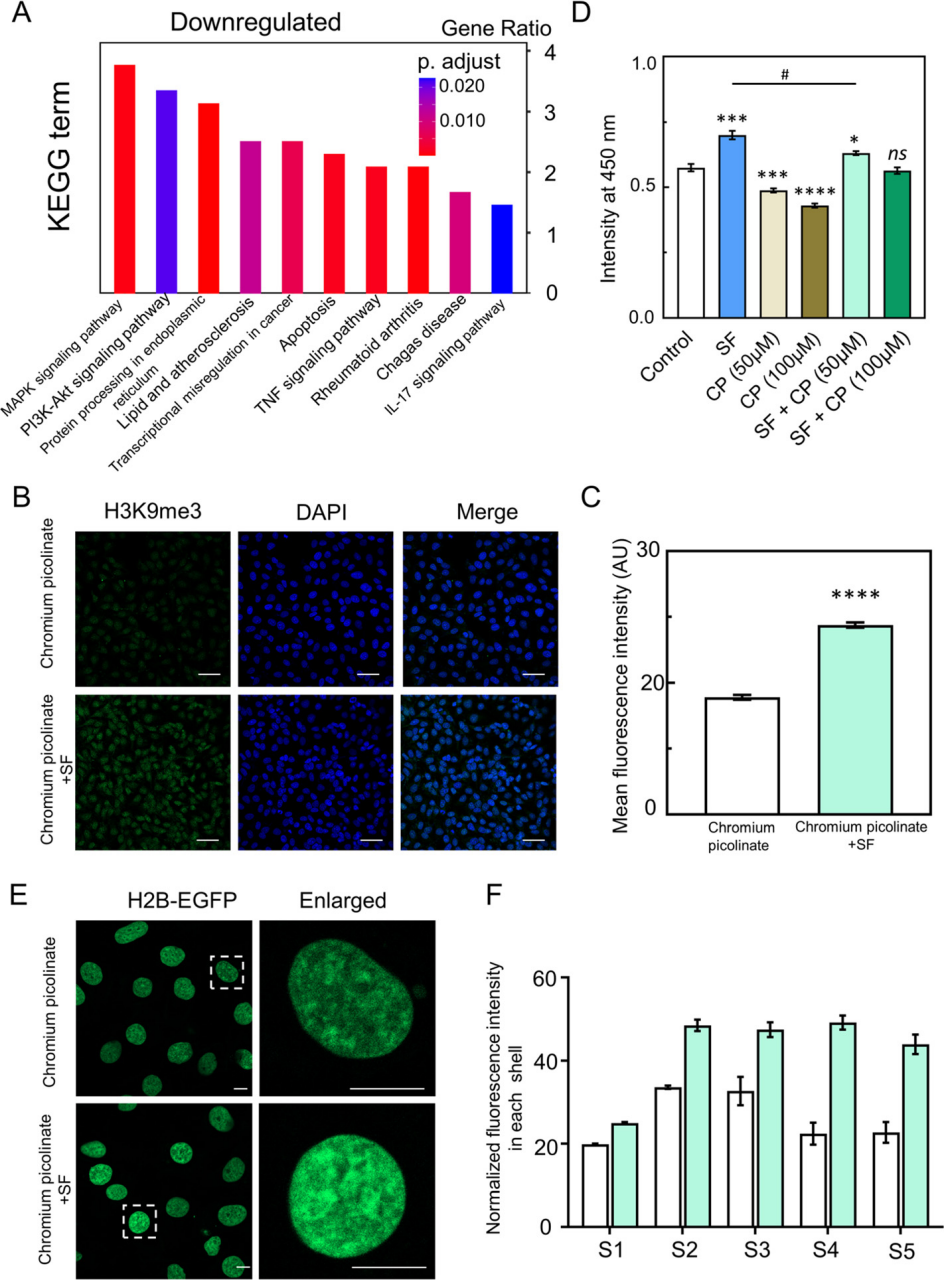
SF promotes EC proliferation through downregulation of the MAPK signaling pathway. (a) KEGG pathway analysis was conducted for the downregulated genes. (b) Immunofluorescence staining was used to detect the levels of H3K9me3 in the chromium picolinate (50 *μ*M) and chromium picolinate-treated SF groups (scale bar = 50 *μ*m). (c) The fluorescence intensity of H3K9me3 in each group was analyzed and compared (n ≥100). (d) Proliferation of HUVECs cultured with chromium picolinate (CP) and chromium picolinate treated with SF for 2 days. (e) H2B-eGFP transgenic HUVECs were treated with chromium picolinate (50 *μ*M) or chromium picolinate treated with 0.4% SF solution for 48 h (scale bar = 10 *μ*m). (f) Normalized fluorescence intensity of H2B-eGFP in each shell in chromium picolinate-treated cells (n = 30) and both chromium picolinate- and SF-treated cells (n = 32). The mean ± SEM is presented based on more than three separate experiments. Compared with control, statistical significance is indicated by ^*^p ≤ 0.05, ^**^p ≤ 0.01, ^***^p ≤ 0.001, and ^****^p ≤ 0.0001. Compared with the SF-treated group, statistical significance is indicated by #p ≤ 0.05. The “ns” indicates no statistical significance. The statistical significance was estimated using a two-tailed t-test.

Next, we found that SF solution treatment downregulated the MAPK signaling pathway in EC through KEGG pathway enrichment analysis [Fig. 5(a)]. To further confirm the involvement of MAPK signaling in SF-induced EC proliferation effect, chromium picolinate, an activator of MAPK was used to activate the MAPK signaling pathway. Immunofluorescence staining analysis showed that the amount of fluorescence of H3K9me3 in SF-treated EC was significantly reduced by chromium picolinate treatment (50 *μ*M) [Figs. 5(b) and 5(c)]. Next, we observed that chromium picolinate treatment inhibited EC proliferation and also reduced the SF solution-promoted EC proliferation [Fig. 5(d)]. These results confirmed that the activation of the MAPK signaling pathway decreased proliferative activity and H3K9me3 levels in SF-treated EC. We further investigated whether the downregulation of MAPK in SF-treated EC was related to chromatin reorganization. We observed that EC treated with both SF and chromium picolinate exhibited an accumulation of chromatin at the nuclear inner cavities when compared to treatment with chromium picolinate alone [Figs. 5(e) and 5(f)]. We also observed that the chromatin organization of EC treated with chromium picolinate and SF displayed an accumulation of H3K9me3-marked chromatin at the nuclear inner cavities when compared to the chromium picolinate treatment alone (Fig. S9). These observations revealed that SF treatment increased proliferative activity, H3K9me3 level, and chromatin reorganization through downregulation of the MAPK signaling pathway in EC.

### F. SF increases cell survival by regulating the H3K9me3 level in oxidative and inflammatory microenvironments

**FIG. 6.**
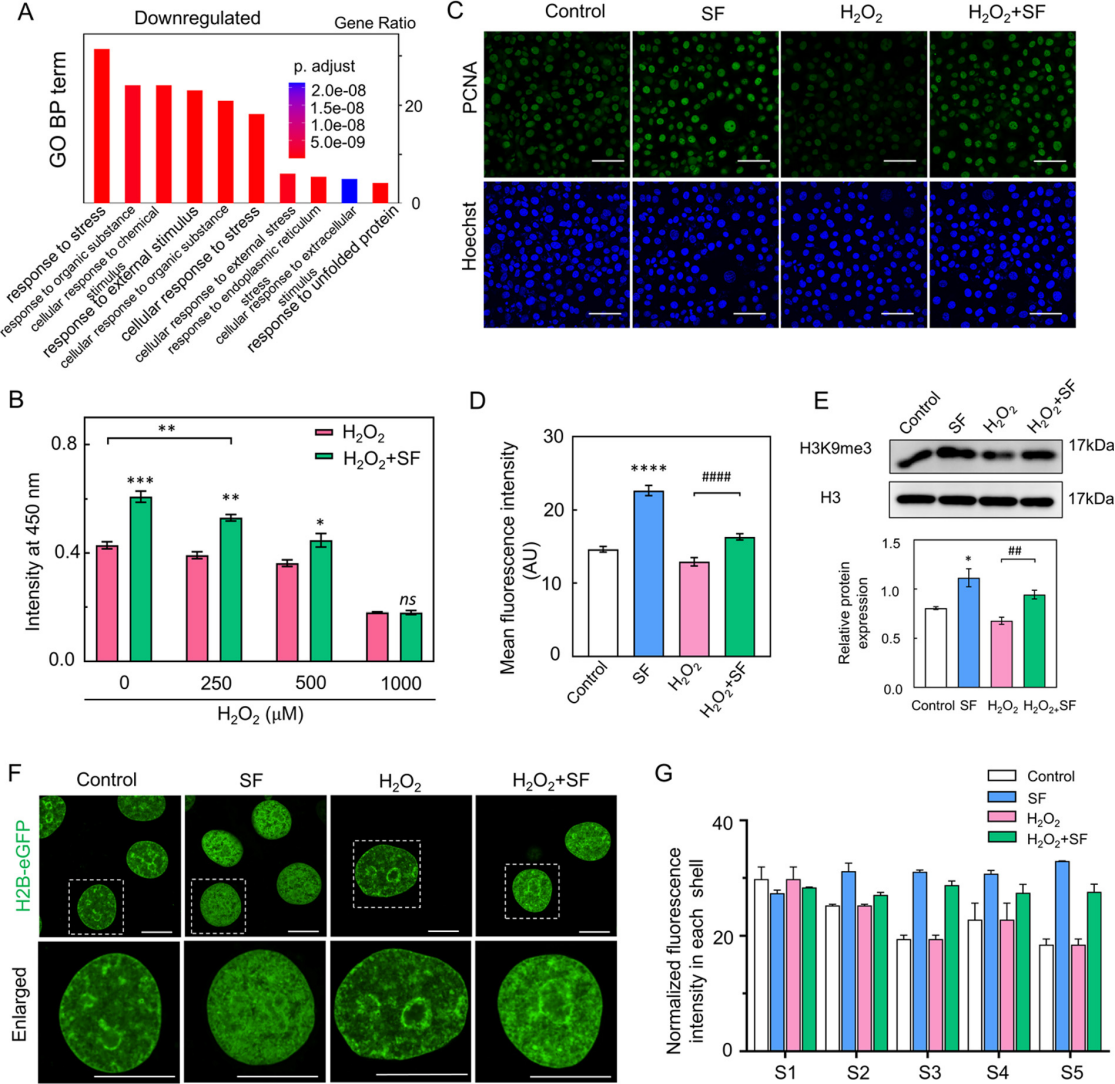
SF increases cell survival by regulating H3K9me3 levels and chromatin reorganization against oxidative insult. (a) KEGG pathway analysis was conducted for the downregulated genes. (b) Pretreatment with the indicated concentration of H_2_O_2_ (250–1000 *μ*M) for 1 h, followed by incubation with 0.4% SF for 24 h. (c) Immunofluorescence staining was used to detect the expression levels of PCNA in the control, H_2_O_2_, and H_2_O_2_-treated SF groups (scale bar = 50 *μ*m). (d) The fluorescence intensity of PCNA in each group was analyzed and compared (n ≥100). (e) The levels of H3K9me3 in the control, SF, H_2_O_2_, and H_2_O_2_-treated SF groups were analyzed by western blot. (f) H2B-eGFP transgenic HUVECs were treated with DPBS, SF, H_2_O_2,_ or H_2_O_2_-treated SF for 12 h (scale bar = 10 *μ*m). Compared to H_2_O_2_ treatment, SF and H_2_O_2_ treatment strongly promoted the localization of H2B-marked overall chromatin at the nuclear center and the formation of large chromatin domains. (G) Normalized fluorescence intensity of H2B-eGFP in each shell in the control, H_2_O_2_, and H_2_O_2_-treated SF groups (n = 30). The mean ± SEM is presented based on more than three separate experiments. Compared with control, statistical significance is indicated by ^*^p ≤ 0.05, ^**^p ≤ 0.01, ^***^p ≤ 0.001, and ^****^p ≤ 0.0001. Compared with the H_2_O_2_-treated group, statistical significance is indicated by ###p ≤ 0.001. The “ns” indicates no statistical significance. The statistical significance was estimated using a two-tailed t-test.

EC faces the challenges of oxidative and inflammatory microenvironments in vascular therapy and tissue engineering. Our investigation aimed to determine whether SF could protect EC under these conditions. According to the KEGG pathway analysis, compared to the control treatment, SF treatment resulted in the downregulation of genes associated with the TNF signaling pathway and the IL-17 signaling pathway [Fig. 5(a)]. The GO analysis also showed a significant downregulation of genes related to the response to stress in SF-treated cells compared to controls [Fig. 6(a)]. To assess EC proliferation in an oxidative microenvironment, cells were pretreated with various concentrations of H_2_O_2_ for 1 h and then incubated with 0.4% SF for 6 h. Cell proliferation was suppressed in the low concentration of H_2_O_2_ (250 *μ*M). Treatment with both SF and H_2_O_2_ significantly increased cell survival when compared to H_2_O_2_ treatment alone (Fig. S10).

Subsequently, a long-term SF treatment assay was performed in H_2_O_2_-treated EC. The cells were pretreated with H_2_O_2_ for 1 h, followed by incubation with 4% SF for 24 h. SF solution significantly increased H_2_O_2_-treated EC proliferation compared to the H_2_O_2_ treatment alone [Fig. 6(b)]. However, no difference was observed between cells treated with H_2_O_2_ or SF and cells treated with H_2_O_2_ alone at a relatively high concentration (1000 *μ*M), likely because of the high concentration causing cell death. Based on these results, 250 *μ*M H_2_O_2_ was selected for subsequent experiments. To confirm the effects of SF on the survival of H_2_O_2_-treated HUVECs, we assessed the expression levels of the inflammatory marker intercellular adhesion molecule-1 (ICAM-1).^20^ The ICAM-1 expression levels were significantly increased in cells treated with H_2_O_2_ alone. However, compared with cells treated with H_2_O_2_ alone, cells treated with H_2_O_2_ and SF exhibited significantly attenuated ICAM-1 expression (Fig. S11). To investigate cell viability and the effects of SF on H_2_O_2_-treated HUVECs, we examined the protein levels of PCNA in H_2_O_2_-treated HUVECs for 24 h. The expression levels of PCNA in HUVECs treated with H_2_O_2_ alone were significantly decreased, whereas cells treated with H_2_O_2_ and SF showed significantly increased PCNA expression compared to treatment with H_2_O_2_ alone [Figs. 6(d) and 6(e)]. Interestingly, the SF treatment significantly increased H_2_O_2_-treated EC proliferation compared to the H_2_O_2_ treatment alone [Figs. 6(b)–6(d)]. These findings demonstrated that SF treatment is sufficient to rescue cell survival against H_2_O_2_-induced ROS insult.

Furthermore, we evaluated the expression of H3K9me3 in H_2_O_2_-treated HUVECs using western blot analysis. The level of H3K9me3 increased in cells treated with H_2_O_2_ and SF compared to that in cells treated with H_2_O_2_ alone [Fig. 6(e)]. We also analyzed chromatin organization in H2B-eGFP HUVECs after H_2_O_2_ treatment. In contrast to the organization observed with H_2_O_2_ treatment alone, the chromatin organization of HUVECs treated with H_2_O_2_ and SF displayed an accumulation of H2B-marked overall chromatin at the nuclear inner cavities [Figs. 6(f) and 6(g)]. Taken together, these results suggest that SF can suppress H_2_O_2_-induced inflammation and promote cell survival by regulating chromatin organization and H3K9me3 levels.

## III. DISCUSSION

The primary objective of this study was to elucidate the role of SF in regulating chromatin organization in EC and the underlying epigenetic mechanisms involved. Through a comprehensive series of systematic investigations, our study has provided novel insights into the epigenetic processes and chromatin organization that underlie the ability of SF to promote EC proliferation. Our study yielded several key findings: (i) The level of H3K9me3 was increased in EC treated with SF. Specifically, the use of a well-known HDAC inhibitor, TSA, to reduce H3K9me3 levels can suppress cell proliferation in EC treated with SF. (ii) SF induces changes in the nucleus and chromatin reorganization in EC. (iii) SF solution enhances EC growth and proliferation through downregulation of the MAPK signaling pathway. (iv) SF promotes EC survival and proliferation after oxidative stress by upregulation of the H3K9me3 level and chromatin reorganization.

SF treatment promotes EC proliferation through increased H3K9me3 levels. Early studies showed that SF can promote cell proliferation and growth.^2,21,22^ When coated on a tissue culture plate and biphasic calcium phosphate scaffold, the SF solution was found to promote cell proliferation.^23^ The present study demonstrated that the SF solution promotes EC cell proliferation (Fig. 1). The higher concentration of SF (3.2%–6.4%) did not enhance a more pronounced proliferation. Early evidence revealed that protein concentration can directly affect the solution viscosity, surface tension, and shear rate.^5,24^ So, the varying concentrations of SF solution may impart disparate solution properties, which could potentially exert different influences on the proliferation of EC. SF treatment promoted EC divisions and increased the protein expression of PCNA in EC. This result is in agreement with early studies where SF promotes NIH3T3 cell proliferation.^2,25^ Therefore, we hypothesized that the SF solution promotes EC proliferation and activates particular epigenetic mechanisms. To assess this hypothesis, RNA-seq analysis was utilized to explore differently expressed genes in HUVECs after treatment with SF. Gene ontology analysis showed significant upregulation of the genes related to the cell cycle process in SF-treated EC compared to controls. SF treatment primarily potentiated the methylation of H3K9-related genes. Western blot and immunofluorescence staining analyses confirmed increased levels of H3K9me3 in both HUVECs and bEnd.3 cells after SF treatment (Fig. 2). This result is supported by previous studies where the cells with greater proliferative capacity exhibited higher levels of H3K9me3.^26,27^ We also observed an accumulation of H3K9me3-marked chromatin in the nuclear inner cavities of cells treated with SF compared to the control group. The EC taken after SF treatment showed no significant difference in the expression level of H3K9me2 when compared to the control groups, which is consistent with a previous report.^16^ Inhibition of the H3K9me3 level by TSA (a histone deacetylase inhibitor) reduced SF solution-promoted EC proliferation through attenuation of the H3K9me3 level (Fig. 3). This result is supported by early studies where TSA treatment significantly decreased the H3K9me3 level, resulting in reduced cell proliferation.^19,28^

SF treatment induced notable changes in chromatin reorganization within EC. Early evidence shows that the decreased expression of replication-dependent canonical histones, including H1, H2a, and H3 histone variants, which are associated with chromatin organization, inhibits cell proliferation.^29^ In the present study, SF treatment upregulated genes related to nuclear organization, such as nucleoplasm, chromosome, chromatin, and condensed chromosome. The expression and activity of nuclear organization regulatory proteins induced by SF treatment would contribute to chromatin reorganization in EC. In this study, we demonstrated that the SF solution promotes the accumulation of chromatin in the inner nuclear cavities (Fig. 4). This result is in agreement with studies where increased H3K9me3 levels facilitate chromatin reorganization.^6,26,30^

SF solution promoted EC growth and proliferation through the downregulation of MAPK genes. MAPK signaling pathway regulates the expression of various genes that are involved in multiple biological processes, including chromatin organization,^10,30^ development,^10^ and epigenetic regulation.^10,31^ Early studies showed that the activation of MAPK signaling decreased H3K9me3 levels and proliferative activity.^26,27,30^ In the present study, RNA-seq analysis exhibited significant downregulation of MAPK genes in SF-treated EC compared to the control. The activation of MAPK signaling by chromium picolinate (a p38-MAPK activator) reduced SF-induced EC proliferative activity and H3K9me3 levels. The chromatin organization of EC treated with chromium picolinate and SF displayed an accumulation of H2B-marked chromatin at the nuclear inner cavities when compared to the chromium picolinate treatment alone. These findings demonstrated that SF promoted EC proliferation through the inhibition of MAPK signaling-mediated upregulation of H3K9me3 levels (Fig. 5).

SF promotes EC survival and proliferation after oxidative stress by chromatin reorganization and regulating H3K9me3 levels (Fig. 6). KEGG pathway analysis revealed that SF treatment downregulated genes related to the TNF and IL-17 signaling pathways. Gene ontology analysis showed a significant downregulation of genes related to the response to stress in SF-treated cells compared to controls. This result is in agreement with an early study where SF suppressed inflammatory reactions and counteracted oxidative stress.^32^ In the present study, the SF solution significantly increased H_2_O_2_-treated EC proliferation compared to the H_2_O_2_ treatment alone. SF solution treatment increased the proliferative activity and PCNA levels and decreased ICAM-1 levels after oxidative stress in EC. We next attempted to determine whether the SF-regulated H3K9me3 level affects cell growth and proliferation in EC after oxidative stress. Early studies have demonstrated that low concentration (200 μM) H_2_O_2_ induced oxidative stress and decreased cell survival, but did not change H3K9me3 levels.^33^ The present study demonstrated that the SF solution increased H3K9me3 levels and promoted chromatin reorganization in EC after oxidative stress. The differences between our results and their observations might be due to the differences in the H_2_O_2_ treatment time and we determined the H3K9me3 level after SF treatment for 24 h. From this evidence, we speculated that SF promotes EC survival and proliferation after oxidative stress by organizing chromatin and regulating the H3K9me3 level.

In summary, this study investigated that SF solution increased proliferative activity, H3K9me3 level, and chromatin organization through downregulation of the MAPK signaling pathway. The results also showed that SF promoted EC survival and proliferation in the presence of an oxidative microenvironment through the upregulation of the H3K9me3 level and chromatin reorganization. Our findings shed new light on histone modification and its potential advantages in the realm of silk-based biomaterials for tissue engineering and regenerative medicine.

## IV. METHODS

### A. Preparation of SF solution

SF was extracted from *Bombyx mori* cocoons sourced from the Northwest Sericulture Base in Shaanxi, China. The SF solution was prepared following the established protocol detailed in prior studies.^2,34^ To eliminate sericin proteins, 5 g of silkworm cocoon was cut into small fragments and subjected to a 30-min boiling process in 1 liter of water containing 5 g/l of sodium carbonate (Na_2_CO_3_; Sangon Biotech, Shanghai, China) at 95 °C; this process was repeated three times. Following degumming, the silk fibers were dried in a 60 °C oven overnight. These fibers were subsequently dissolved in Ajisawa's reagent (CaCl_2_:CH_3_CH_2_OH:H_2_O molar ratio = 1:2:8; Sangon Biotech, Shanghai, China) at 60 °C for 4 h. The SF solutions were acquired through dialysis employing a 3500 cellulose membrane with a molecular weight cutoff (Biosharp, Hefei, China) within 5 l of ultrapure water for 3 days. The water was replaced every 3 h to eliminate salt. After completion of dialysis, the solutions were centrifuged twice at 5000 rpm for 5 min at 4 °C to eliminate any residual silk aggregates. The final concentration of the SF solution was approximately 10% (wt/vol), as ascertained by weighing the remaining solid posturing. Ultimately, the silk fibroin was filtered using a 0.2 *μ*m filter and stored at 4 °C for all subsequent experiments.

### B. Cell culture

The human umbilical vein endothelial cell (HUVEC) line was obtained from ScienCell Research Laboratories. Mouse brain endothelial bEnd.3 cells (bEnd.3) were obtained from the Baidi Biotechnology Co., Ltd (Zhejiang, China). Cells were cultivated in DMEM (Gibco, Grand Island, NY, USA) supplemented with 10% fetal bovine serum (FBS) and 1% penicillin–streptomycin antibiotic solution (P/S). HUVECs were incubated in 6 cm culture dishes at 37 °C in an atmosphere containing 5% carbon dioxide (CO_2_) with 95% humidity. The culture medium was replaced every 2 days. Once the confluence reached 80%–90%, HUVECs were expanded via trypsinization using 0.25% trypsin containing 0.02% EDTA and subsequently resuspended in a fresh growth medium.

### C. Cell proliferation assay

To assess the effect of SF treatment on EC proliferation and viability, we employed the cell counting kit 8 (CCK-8; abbkine; Wuhan, Hubei, China) assay in accordance with the manufacturer's guidelines.^2^ HUVECs were initially seeded at a density of 5 × 10^3^ cells per well in 96-well plates (Jet Biofil, Guangzhou, Guangdong, China) and were allowed to incubate overnight at 37 °C in a 5% CO_2_ incubator. These cells were cultured in DMEM supplemented with 10% FBS and 1% P/S. After the removal of the medium, 100 μL of fresh DMEM containing varying concentrations of SF (0, 0.2%, 0.4%, 0.8%, 1.6%, 3.2%, and 6.4%) was added to the HUVECs, after which the cells were cultivated for periods of 1, 2, and 3 days. Following the incubation period, the SF treatment medium was aspirated, and the cells were cultured with 100 *μ*l of fresh FBS-free medium containing CCK-8 reagent (at a ratio of 10:1) for 30 min at 37 °C. Ultimately, the optical density was determined at 450 nm using a microplate reader (Bio-Tek, Winooski, VT, USA).

### D. Construction of the H2B-eGFP stable cell line and imaging

The H2B-eGFP HUVEC line was established through the transduction of HUVECs with retroviral constructs encoding H2B-eGFP variants. Subsequently, these cells were sorted based on eGFP expression using flow cytometry (Sony Biotechnology, Tokyo, Japan). Following sorting, the cells were cultured in 24-well plates within a CO_2_ chamber maintained at 37 °C. These cells were subjected to treatment with 0.2% or 0.4% SF for 0, 12, 24, or 48 h. The resultant samples were then subjected to imaging through laser scanning confocal microscopes (Nikon AX, Japan) (Zeiss LSM980, Germany).

We performed quantitative analyses of chromatin changes following the established protocol detailed in previous studies.^35^ We measured H2B-eGFP plot profiles across the nucleus diameter of the equatorial focal plane with ImageJ software. The individual plot profiles were normalized on the average intensity and divided into five shells with equal distance from the nuclear periphery to the interior.

### E. Flow cytometry analysis

For the proliferation assay, HUVECs were labeled with CellTrace Violet (CTV; Invitrogen, Carlsbad, CA, USA) following the manufacturer's instructions.^17^ Subsequently, the labeled cells were seeded in 6-well culture plates (Corning, NY, USA) and allowed to incubate overnight at 37 °C in a 5% CO_2_ incubator. Following this incubation period, the cells were exposed to either 0.2 or 0.4 (wt. %/vol. %) SF or Dulbecco's phosphate-buffered saline (DPBS) as a control. After two days, the labeled cells were trypsinized and collected in a fresh growth medium. Flow cytometry analysis was performed using Beckman Coulter equipment (Brea, CA, USA), and FlowJo software was used to analyze the cell proliferation data.

### F. Scratch assays of cell migration

ECs were initially plated in 6-well culture plates and maintained at 37 °C in a 5% CO_2_ incubator. Once the cells reached confluence, a scratch was generated using a 20 *μ*l micropipette tip, followed by two washes with DPBS solution. Subsequently, each well was replenished with fresh DMEM supplemented with either 0.2% or 0.4% SF, while DPBS served as the control. The cells were then incubated for a period of up to 12 h or 24 h at 37 °C in a 5% CO_2_ incubator. Microscopy images were captured at both the 0-h and 12- or 24-h time points using a phase-contrast microscope. To assess cell migration, cell-free areas in the microphotographs were quantified using ImageJ software for comparative analysis.

### G. Immunofluorescence staining and quantification

For immunofluorescence experiments, HUVECs were cultured on a 35 mm confocal dish (Biosharp, Hefei, China). The cells were fixed with 4% paraformaldehyde (PFA; Biosharp, Hefei, China) for 15 min at room temperature, followed by three washes with phosphate-buffered saline (PBS; BBI, Shanghai, China) for 5 min each. Subsequently, permeabilization was carried out using 0.25% Triton X-100 (Biosharp, Hefei, China) in PBS for 10 min at room temperature, followed by another three washes with PBS. The cells were then blocked with 5% goat serum (Invitrogen, Grand Island, NY) in PBS at room temperature for 2 h. Following blocking, primary antibodies were applied to the cells in PBS (with 1% goat serum), which were subsequently incubated overnight at 4 °C. The primary antibodies utilized in this study included anti-H3K9me3 antibodies (CST, 13969, diluted 1:1000), anti-H3K9me2 antibodies (CST, 4658, diluted 1:1000), anti-PCNA antibodies (Santa Cruz, sc-56, diluted 1:500), and anti-ICAM-1 antibodies (Proteintech, 16174-1-AP, diluted 1:800). After washing three times for 5 min each with PBST (0.1% Triton X-100 in PBS), the cells were incubated with secondary antibodies diluted in blocking buffer for two hours at room temperature. The secondary antibodies used were goat anti-mouse IgG Alexa 488 (CST, 4408S, diluted 1:1000), goat anti-rabbit IgG 594 (CST, 8889S, diluted 1:1000) and goat anti-rabbit IgG 488 (Proteintech, SA00013-2, diluted 1:1000). After another three washes with PBS, Hoechst 33342 (Thermo Fisher Scientific, diluted 1:1000), or DAPI (Thermo Fisher Scientific, diluted 1:1000) was used to stain the cell nuclei for 15 min. Subsequently, the cells were subjected to three additional 5-min washes with PBS before being mounted with the mounting medium from Thermo Fisher Scientific. The samples were imaged utilizing a laser scanning confocal microscope (Olympus FV3000, Japan) (Nikon AX, Japan). Relative fluorescence units were quantified using ImageJ software.

### H. Western blot analysis

The cultured cells were resuspended in 100 *μ*l of RIPA lysis and extraction buffer (Pierce, Rockford, IL, USA) supplemented with 1 mM phenylmethylsulfonyl fluoride (PMSF). Subsequently, the cell lysates were sonicated to fragment genomic DNA and subjected to centrifugation at 15 000 × g for 15 min at 4 °C. The protein concentration in the resulting supernatants was determined using a bicinchoninic acid (BCA) protein assay. The denatured proteins were then separated on an SDS–polyacrylamide gel and subsequently transferred onto polyvinylidene difluoride membranes (Millipore, Billerica, MA, USA). Following the transfer, the membranes were blocked with 5% nonfat milk for 2 h at room temperature. Subsequently, the sections were incubated overnight at 4 °C with primary antibodies diluted 1:1000 in 5% nonfat milk in PBS. The primary antibodies used were anti-H3K9me3 (CST, 13969; diluted 1:1000) and anti-PCNA (Santa Cruz, sc-56; diluted 1:500). The membranes were subjected to three washes with 0.1% Tween-20 in Tris-buffer saline and subsequently incubated with horseradish peroxidase-conjugated IgG secondary antibodies (CST, 7074 or 7076, diluted 1:1000) at room temperature for 2 h. Finally, the immunoreactive bands were visualized utilizing an Omni-ECL™ Femto Light Chemiluminescence Kit (EpiZyme, Shanghai, China) with a ChemiDoc MP Imaging System (Bio-Rad, Singapore). The gray values of the blots were quantified using ImageJ software.

### I. RNA sequencing

RNA was extracted from HUVECs treated with 0.4% SF on day 2 utilizing Total RNA Kit I reagent (Omega Bio-Tek, GA, USA) following the manufacturer's guidelines. The integrity of the isolated RNA was assessed using an Agilent 2100 Bioanalyzer. Subsequently, library preparation and sequencing were performed on the DNESEQ-T7 platform, generating 150 bp paired-end reads. To process the RNA reads Trim Galore v0.6.10 was used to eliminate adapters and filter out low-quality reads. The processed reads were subsequently aligned to the GRCh38/hg38 reference genome utilizing STAR v2.7.3a software.^36^ Expression quantification was carried out using the feature counts (subread v2.0.0) tool^37^ with gene annotation obtained from *Homo sapiens* Ensembl v110. Differential expression analysis was conducted employing the DESeq2 v1.32.0 tool.^38^ Genes that exhibited a fold change greater than 1.2 and an adjusted *p*-value less than 0.05 were considered differentially expressed. Furthermore, functional annotation enrichment analysis was performed using the bioinformatics resources provided by the Database for Annotation, Visualization, and Integrated Discovery (DAVID).^39^

### J. TSA treatment and antioxidative activity assay

HUVECs were subjected to TSA treatment following established protocols detailed in prior studies.^9,16,19^ The cells were initially seeded at a density of 5 × 10^3^ cells per well in 96-well plates and allowed to incubate overnight. Subsequently, the cells were exposed to different conditions, including 0.4% SF, 100 nM TSA, or a combination of 100 nM TSA with 0.4% SF, for 2 days. A negative control sample was treated with dimethyl sulfoxide (DMSO). Cell survival was assessed using the CCK-8 assay.

The objective of the present study was to evaluate the influence of SF on endothelial cell death induced by oxidative damage caused by varying concentrations of hydrogen peroxide (H_2_O_2_). HUVECs were seeded at a density of 5 × 10^3^ cells per well in 96-well plates. These cells were pretreated with H_2_O_2_ at concentrations of 0, 250, 500, and 1000 *μ*M for 1 h. Subsequently, the cells were cultured in fresh medium containing 0.4% (wt/vol) SF for 24 h at 37 °C in a 5% CO_2_ incubator. Following the treatment, the SF-containing medium was removed, and the cells were cultured in 100 *μ*l of fresh medium devoid of FBS. Cell survival was determined utilizing the CCK-8 assay.

### K. Chromium picolinate treatment assay

HUVECs were subjected to chromium picolinate treatment following established protocols detailed in prior studies.^40^ The cells were initially seeded at a density of 5 × 10^3^ cells per well in 96-well plates and allowed to incubate overnight. Subsequently, the cells were exposed to different conditions, including 0.4% SF, chromium picolinate (50,100 *μ*M), or a combination of chromium picolinate with 0.4% SF for 2 days. A negative control sample was treated with dimethyl sulfoxide (DMSO). Cell survival was assessed using the CCK-8 assay.

### L. Statistical analysis

The data are expressed as the mean ± standard error of the mean (SEM). All the statistical analyses were conducted using GraphPad Prism 8.0 software for Windows. Differences between the two groups were evaluated utilizing Student's t-test. Statistical significance was denoted as ^*^p < 0.05, ^**^p < 0.01, ^***^p < 0.001, and ^****^p < 0.0001, and ns indicates no significance.

## SUPPLEMENTARY MATERIAL

See the supplementary material for supplementary material figures (Figs. S1–S11).

## Data Availability

The data that support the findings of this study are available within the article and its supplementary material.
